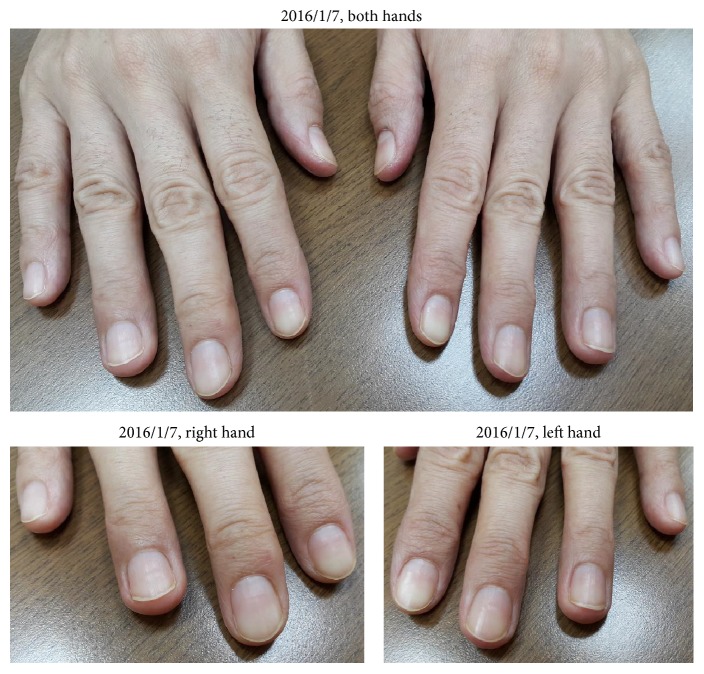# Chinese Medicine Treatment for Afatinib-Induced Paronychia

**DOI:** 10.1155/2017/7327359

**Published:** 2017-08-29

**Authors:** Pei-Yuu Yang, Chen-Jei Tai

**Affiliations:** ^1^Department of Traditional Chinese Medicine, Taipei Medical University Hospital, Taipei 11031, Taiwan; ^2^Department of OB/GYN, School of Medicine, College of Medicine, Taipei Medical University, Taipei 11031, Taiwan

## Abstract

Afatinib (Gilotrif™) is widely used to treat patients with mutant activating epidermal growth factor receptor- (EGFR-) dependent lung adenocarcinoma; however, it has various adverse side effects. Here, we report a patient with afatinib-induced paronychia. After Chinese medicine treatment with the well-known anticancer Chinese herbs, Jen-Ren-Hwo-Minq-Saan, and decoction of Ban-Zhi-Lian* (Scutellaria barbata)* with Bai-Hua-She-She-Cao (*Hedyotis diffusa* Willd), patient's condition was significantly improved. This shows that these Chinese medicines can not only be used in cancer treatment but also be used in the afatinib-induced paronychia.

## 1. Introduction

Lung cancer is one of the most commonly diagnosed cancers and is the leading cause of cancer deaths around the world. Non-small-cell lung cancer (NSCLC) accounts for about 80% of all lung cancers and is often diagnosed at the late stage [1]. NSCLC comprises non-squamous-cell carcinoma (i.e., adenocarcinoma, large-cell carcinoma, and other cell types) and squamous cell carcinoma [1]. Some patients with lung adenocarcinoma have common activating epidermal growth factor receptor (EGFR) mutations: deletions in exon 19 and exon 21 L858R substitution mutations [2]. Thus, targeting EGFR has become an important strategy in the treatment of NSCLC. Specifically, oral afatinib (Gilotrif) is approved for the first-line treatment for patients who have metastatic NSCLC with EGFR exon 19 deletions or exon 21 (L858R) substitution mutations as detected by an FDA-approved test. The recommended dosage of afatinib is 40 mg once daily; treatment should be continued until disease progression or until afatinib is no longer tolerated [3]. Adverse events are generally manageable with dose reductions and delays. Among afatinib recipients, the most common treatment-related adverse events (all grades, incidence of >10%) included diarrhea (95.2% of patients), rash/acne (89.1%), stomatitis/mucositis (72.1%), paronychia (56.8%), dry skin (29.3%), decreased appetite (20.5%), pruritus (18.8%), nausea (17.9%), fatigue (17.5%), vomiting (17.0%), epistaxis (13.1%), and cheilitis (12.2%) [4]. Many people interrupt their therapies because of the seriously adverse side effects. Therefore, reducing the adverse side effects will greatly improve patients' outcome. This study reports that Chinese medicine can cure paronychia induced by afatinib.

## 2. Case Report

A 42-year-old female was diagnosed with adenocarcinoma in her lower and middle lobes of her right lung and bone, liver, and brain metastasis in 2015/8. She started taking afatinib from 2015/8/18. Then paronychia, oral ulcer, and diarrhea symptoms appeared.

**Figure 1:**
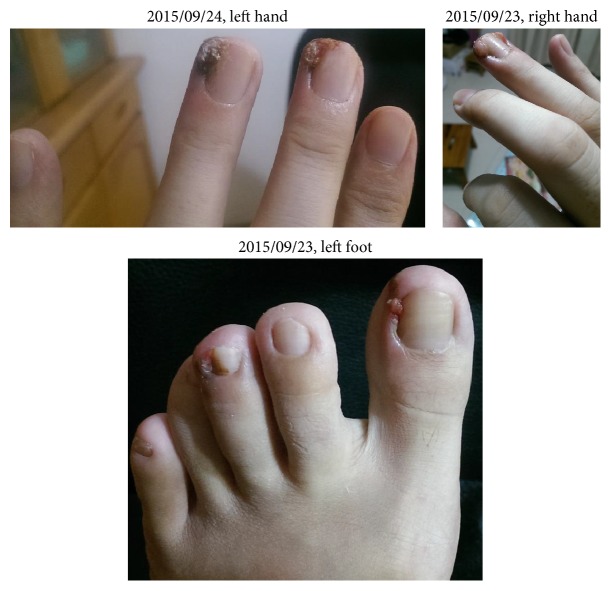


We can see paronychia on both of her hands and left foot in the photos in 2015/9. From the pictures she took, we can see paronychia on the lateral side of the middle and ring finger of her left, both sides of the ring finger of her right, ulcers, and hypertrophic granulation tissue on the lateral side of the first and middle toes of her left foot (Figure 1).

The patient came to Chinese Medicine Clinics on 2015/10/1. Her chief complaint was paronychia with pain and tissue fluid exudation. We prescribed to her concentrated scientific herbal medicine of Jen-Ren-Hwo-Minq-Saan and Chinese herb decoction of Ban-Zhi-Lian with Bai-Hua-She-She-Cao. The dosage of concentrated scientific herbal medicine, Jen-Ren-Hwo-Ming-Sann, was 6 grams per day in 3 divided doses taken after each meal. Besides, the dosage of crude drugs in each pack of decoction of Ban-Zhi-Liagn with Bai-Hua-She-She-Cao was 9.375 grams each. The patient had to take three packs every day. She took all these medicine above during the therapy.

To monitor whether the Chinese medicines affect the afatinib treatment, a blood test was issued. Her blood test report on 2015/10/19 was as follows: CEA: 134, WBC: 6520, GOT: 28, and GPT: 24.

**Figure 2:**
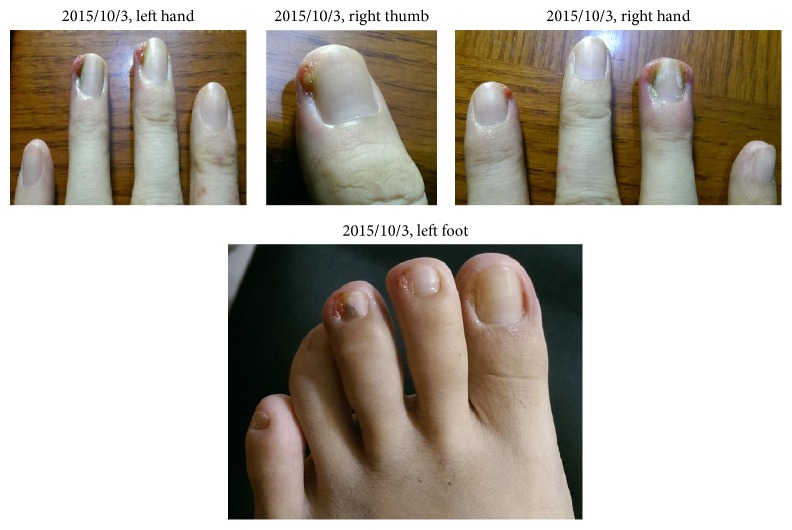


The patient came back to clinic on 2015/10/22 with the pictures she took on 2015/10/3, when she had taken the medicine for 3 days (Figure 2).

**Figure 3:**
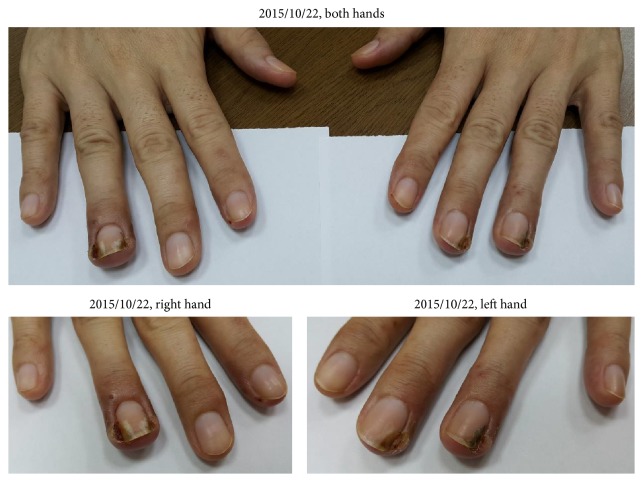


We could observe that the granulation tissue almost disappeared and ulcer had improved, although the wound had not healed yet. Therefore, she continued taking the concentration scientific herbal medicine of Jen-Ren-Hwo-Minq-Saan and decoction of Ban-Zhi-Lian and Bai-Hua-She-She-Cao. The condition of the paronychia was recorded on 2015/10/22 and is shown in Figure 3.

**Figure 4:**
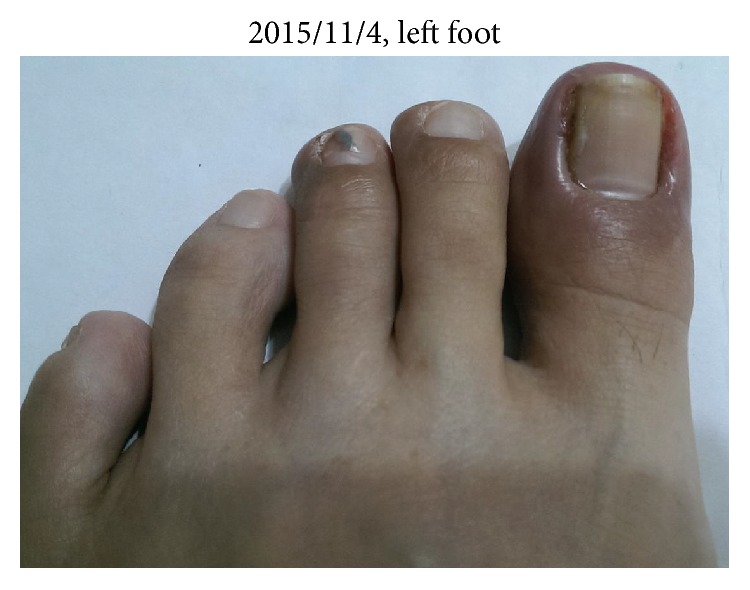


We can find that the wounds on the left foot also continue improving with the pictures taken by patient herself in 2015/11/4 (Figure 4).

**Figure 5:**
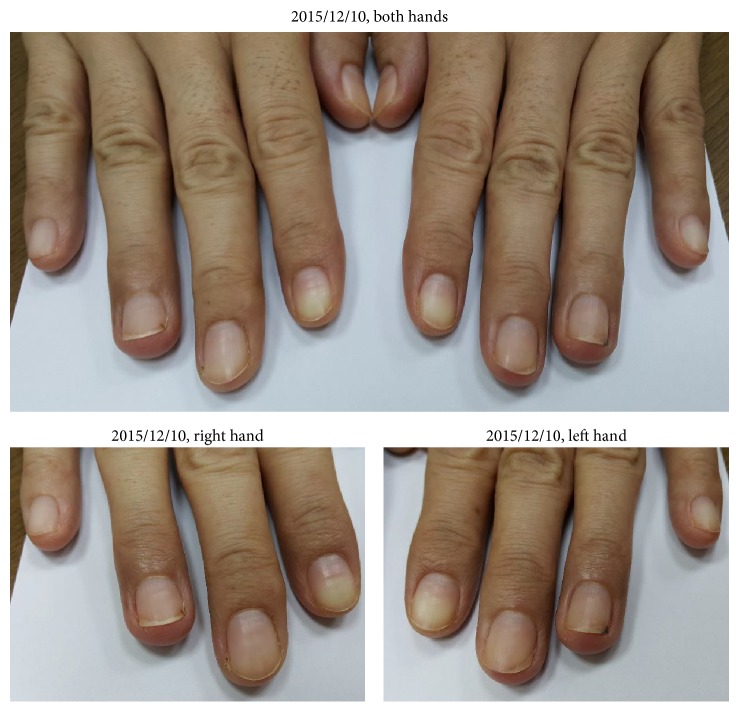


The wounds on both hands were almost cured in the pictures in 2015/12/10 (Figure 5). We advised her to continue the treatment.

The pictures showed that paronychia had been completely cured in 2016/1/7 (Figure 6). And her blood test report on 2016/1/18 was as follows: CEA: 124.4, WBC: 5910, and GOT: 17.

After the treatment of paronychia, she continued taking these medicines for controlling the tumor cells, and the paronychia never returns.

## 3. Discussion

The most tormenting side effect of afatinib for this patient is paronychia. From Figures 1–6, we can easily observe the efficacy of the Chinese medicine treatments. After the treatment, paronychia had almost been cured in two months. Note that the reduction of CEA showed that the Chinese medicine did not influence the effect of afatinib. This patient did not take any antibiotics or use topical medication to deal with paronychia during the treatment of traditional Chinese medicine. Because the patient keeps taking afatinib, we will not discontinue her Chinese medicine treatment even though her paronychia was cured. Therefore, we do not know whether the paronychia will recur after stopping taking Chinese medicine, but it is quite possible.

Zhen Ren Huo Ming Yin, also called Xian Fang Huo Ming Yin, is a traditional Chinese herbal complex medicine. It was often used to treat skin illness, such as skin ulcers, sores, and wounds. Nowadays, researches have shown the efficacy of Zhen Ren Huo Ming Yin on ulcerative colitis [5] and fibrocystic disease of the breast [6].

Bai-Hua-She-She-Cao,* Hedyotis diffusa* Willd, is a well-known Chinese herb. It has neuroprotective constituents [7] and antitumor activity [8].* Hedyotis diffusa* has been proved to inhibit tumor angiogenesis [9] and fight against HepG2 carcinoma cells mediated via apoptosis [10].* Hedyotis diffusa* extract can inactivate human colorectal cancer cells [11] and breast cancer cells [12].

Ban-Zhi-Lian,* Scutellaria barbata*, is a traditional Chinese herbal medicine originated from southern China and is widely used as an anti-inflammatory and diuretic herb. Several studies have indicated that extracts of* Scutellaria barbata* have growth inhibition effects on a number of human cancers. Treatment of lung cancer, digestive system cancers, hepatoma, breast cancer, and chorioepithelioma by* Scutellaria barbata* extracts was reported [13].

In addition, many papers show the efficacy of* Hedyotis diffusa* plus* Scutellaria barbata* on multiple types of cancers.* Hedyotis diffusa* plus* Scutellaria barbata* can induce apoptosis in bladder cancer cells [14] and has been used for postsurgery colon cancer patients in Taiwan [15]. This kind of combined remedy is the core treatment for breast cancer patients in Chinese medicine [16].

## 4. Conclusion

The Chinese medicines that were used to treat paronychia in this report are well-known anticancer drugs. However, the efficacy for these anticancer drugs to treat paronychia induced by the targeted therapy, afatinib, was never reported to our knowledge. With the ability to cure afatinib-induced paronychia, we are confident that, with the combination of Jen-Ren-Hwo-Minq-Saan and decoction of Ban-Zhi-Lian with Bai-Hua-She-She-Cao, we can relieve the patients from severe side effect and reduce the rate for patients from dropping the afatinib treatment. Paronychia was almost completely recovered in two months in this case, but more cases are required to realize how long in average should the Chinese medicine be prescribed for paronychia sufferers.

## Figures and Tables

**Figure 6:**